# Urinary neutrophil gelatinase-associated lipocalin (uNGAL) and kidney injury molecule-1 (uKIM-1) as markers of active lupus nephritis

**DOI:** 10.1007/s10067-023-06698-2

**Published:** 2023-07-29

**Authors:** Walaa Hosny Mohammed Ibrahim, Alaa AbdelAziz Sabry, Ahmed Raafat Abdelmoneim, Hamdy Fouad Ali Marzouk, Rasha Mahmoud AbdelFattah

**Affiliations:** 1 Nephrology Unit, Internal Medicine Department, Faculty of Medicine, Assiut University, Assiut, Egypt; 2 Nephrology Unit, Internal Medicine Department, Faculty of Medicine, Mansoura University, Mansoura, Egypt; 3 Clinical Pathology Department, Faculty of Medicine, Mansoura University, Mansoura, Egypt

**Keywords:** Active lupus Nephritis, uNGAL, uKIM-1, Urinary biomarkers

## Abstract

**Background and objectives:**

Despite much research about lupus nephritis, none of the urinary biomarkers has been proven to be truly reflecting lupus nephritis activity, response to treatment, or prognosis. We aimed to study urinary biomarkers in lupus nephritis and test their relation to kidney damage.

**Patients and methods:**

Forty patients with systemic lupus erythematosus (SLE) were divided into two graoups: (1) lupus nephritis group with biopsy-proven proliferative lupus nephritis (classes III and IV) and who did not receive immunosuppressive drugs within the preceding 3 months except for glucocorticoids and (2) lupus non-nephritis group with SLE patients without any renal manifestation. We assessed disease activity by the SLE disease activity index. uNGAL, uKim-1, uNGAL to urinary creatinine excretion (mg/dl), and uKim-1 to urinary creatinine excretion were measured in random spot urine samples at the time of renal biopsy and 6 months after the induction therapy.

**Results:**

The LN group before treatment showed higher levels of uNGAL and uKIM-1 (*P*-value < 0.001). ROC analysis showed that uNGAL at level of > 59 has a 95 % sensitivity, a 100 % specificity, and an AUC = 0.996 in the ability to diagnose LN. While the uKIM-1 ROC showed that at level of > 1.6, it has an 85 % sensitivity, an 80 % specificity, and an AUC = 0.919. uNGAL and uKIM levels were significantly lower after treatment (*P*-value < 0.001). No significant correlations were found between urinary markers before and after treatment with other clinical, inflammatory, and serological markers of lupus nephritis.

**Conclusion:**

uNGAL, uKIM, uNGAL/Creat ratio, and uKIM/Creat ratio can be used as a predictor and a marker of disease activity for lupus nephritis.

**Table:** 

**Key Points** *• Renal biopsy is the current standard for diagnosis of lupus nephritis and none of the urinary biomarkers has been fully concluded to have a diagnostic power to reflect the activity or the response to treatment.* *• However, based on the finding of the current study, uNGAL, uKIM, uNGAL/Creat ratio, and uKIM/Creat ratio showed significant diagnostic performance and were powerful indices of renal involvement in systemic lupus patients and as markers of disease activity.*

## Introduction

Lupus nephritis affects 30–60% of systemic lupus patients, and it is highly related to increased morbidity and mortality [1]. Ten to fifteen percent of lupus nephritis patients progress to end-stage renal disease (ESRD), and the 5-year survival is about 82% compared to 92 % for those without nephritis [2]. Early treatment for lupus nephritis has a beneficial effect on the progression of lupus nephritis, while late diagnosis is associated with increased rate of ESRD [3].

Renal histopathology is still the gold standard test for the diagnosis of lupus nephritis (LN), and for determining the degree of activity or chronicity of LN [4]. However, renal histopathology has its shortages. Not only it needs an invasive procedure but also it neglects certain diagnoses, for instance, thrombotic microangiopathy, vasculitic lesions, and tubulointerstitial involvement, that can change the plan of management. In addition, renal histopathology cannot be used as a way for monitoring the course of lupus nephritis [5].

Many serological and urinary biomarkers have been studied longtime ago and showed correlation with different histological findings of lupus nephritis [4], for example, but not limited to, monocyte chemoattractant protein-1 (MCP-1) [6], TNF-like weak inducer of apoptosis (TWEAK) [7], neutrophil gelatinase-associated lipocalin (NGAL) [6], and vascular cell adhesion molecule-1 (VCAM) [6, 8].

High levels of NGAL were reported after ischemic or toxic kidney injury in animal models [9], cardiovascular surgery [10], intensive care units [11], post renal transplantation graft dysfunction [12], contrast-induced nephropathy [13], cisplatin-induced nephrotoxicity [5], and Chronic Kidney Disease (CKD) [14, 15].

However, there is a paucity of studies investigated urinary NGAL (uNGAL) as a predictor of LN activity [16, 17, 18], especially in patients with an existing renal disease [19]. Moreover, there is no extensive studying of the uNGAL/creatinine ratio in patients with LN exacerbation or its role in monitoring disease response to therapy [4, 20].

Clinical studies indicate that Urinary Kidney Injury Molecule-1 (uKIM-1) was a sensitive and specific marker of tubular injury [21, 22], especially in lead toxicity [23]. Not better off uNGAL, literature shows the paucity of studies investigated the use of uKIM-1 as a marker of renal injury in lupus nephritis [24].

We hypothesized that uNGAL, uNGAL/creatinine ratio, uKIM-1, and uKIM-1/creatinine ratio can be useful predictor markers of LN activity (classes III and IV) by comparing their levels in patients with active LN versus systemic lupus patients without proven renal involvement and healthy controls.

## Methods

### Study design and ethics

This prospective cohort study with control group was conducted in the Nephrology Unit of the Internal Medicine Department of Mansoura University Hospital between January 2019 and December 2020. The study was conducted according to the Code of Good Practice and the guidelines of the Declaration of Helsinki, 7th revision, 2013. In addition, it was approved by the Medical Ethics Committee of the Faculty of Medicine. A written informed consent was obtained from all participants before being enrolled in the study. Patient privacy and confidentiality were respected throughout the whole process of testing and analyzing data. All collected data remained confidential.

### Study groups

Forty patients with SLE was divided into three groups. The first group were LN patients, who were > 18 years, diagnosed with SLE according to 1997 modified American College of Rheumatology (ACR) criteria with biopsy-proven proliferative LN (classes III and IV), and did not receive immunosuppressive drugs within the preceding 3 months except for glucocorticoids. Patients in this group were at their first nephritis flare and it was their first time to be diagnosed with active lupus nephritis. Elderly > 60 years, pregnant females, patients with severe infection, other immune diseases or other classes of LN, and patients unfit for renal biopsy were all excluded from the study. The second group was lupus non-nephritis patients who were >18 years, diagnosed with SLE, and without any renal manifestation. The third group was the control group comprised of randomly selected health care workers with matched age and sex.

### Patients

All participants have been subjected at enrollment to the following: detailed medical history taking and systemic physical examination. Assessment of disease activity was done by SLE disease activity index (SLEDAI) (Isenberg et al., 2005). Accordingly, patients who had score less than 4 were considered as inactive, score 4–8 = mild disease activity, score 9–12 = moderate disease activity, and score more than 12 = severe disease activity. Minimum score was 0 and maximum score is 105 [25].

### Laboratory investigations

Laboratory investigations included the following: serum creatinine, urine analysis, 24-h urinary protein, complete blood count (CBC), erythrocyte sedimentation rate (ESR), C-reactive protein (CRP), antinuclear antibody (ANA), anti double stranded DNA (antidsDNA), serum complement (C3), (C4), uNGAL to urinary creatinine excretion, uKim-1 to urinary creatinine excretion; urine samples and serological investigations were done 24–48 h before renal biopsy. The uNGAL and uKim-1 were measured in random spot urine samples at the time of the renal biopsy and 6 months after the induction treatment. Ultrasound-guided renal biopsy was performed on patients with active LN in the nephrology department unless contraindicated. The biopsies were evaluated according to the revised International Society of Nephrology/Renal Pathology Society (ISN/RPS) classification of lupus nephritis (Weening et al., 2004b). Based on kidney biopsy, the evaluation of disease activity and chronicity was according to the modified NIH activity and chronicity scoring system (Stokes & D'Agati, 2019). Patients were treated according to the 2012 kidney disease: Improving Global Outcomes KDIGO guidelines.

After 6 months of induction therapy, the group of active LN patients were subjected to the following laboratory investigations: serum creatinine, urine analysis, 24-h urinary protein, CBC, ESR, CRP, ANA, anti-dsDNA, serum C3, and serum C4.

Eleven out of 20 active LN patients received pulse steroid (500 mg methyl prednisone for 3–5 days) plus 500 mg cyclophosphamide biweekly for 3 months as an induction therapy and the other nine patients received pulse steroid therapy plus mycophenolate mofetil MMF (2–3 g for 6 months). The response to the induction therapy was evaluated after 6 months according to KDIGO guideline (2012).

Sterile containers were used to collect urine samples. Any particulates were removed by centrifugation for 15 min at 1000×g at 2–8 °C and assayed immediately or aliquoted and stored at −20°C or −80 °C. Centrifugation was done again before assaying to remove any additional precipitates that may appear after storage. All reagents and samples were brought to room temperature before use. All samples and standard were assayed in uNGAL, and uKIM-1 were quantified by ELISA (mybiosource, Catalog no MBS584559 and MBS700484).

### Statistical analysis

Data were entered and analyzed using IBM-SPSS software (IBM SPSS Statistics 19 for Windows). Qualitative data were expressed as absolute frequency (*N*) and percentage (%), while quantitative data were expressed as mean ± standard deviation (SD) if normally distributed without significant outliers, or median and interquartile range (IQR) if not. For comparing qualitative data, chi-square test was used, while Fisher’s exact test was used when any of the frequencies produced by the null hypothesis are less than 5. The independent-samples *t*-test or its non-parametric equivalent, Mann-Whitney *U* test, was used for the comparison of quantitative data. The paired-samples *t*-test or its non-parametric equivalent, Wilcoxon’s signed-rank test, was used to compare paired data. One-way ANOVA or its non-parametric equivalent, Kruskal-Wallis *H* test, was used to compare multiple groups. Pairwise comparisons were used to properly compare each pair of data. The diagnostic performance of a test or the accuracy of a test to discriminate diseased cases from non-diseased cases is evaluated using receiver operating characteristic (ROC) curve analysis.

## Results

### Before treatment

Personal characteristics, SLE score, and laboratory results of the study group are shown in Table 1. There were no significant differences between the 3 groups in age and gender with a noted female predominance in all groups. The mean of SLEDAI score in the active LN group before treatment was significantly higher (18.5) than that in the non-nephritis group (7.5) (*P*-value <0.001). Serum creatinine was significantly higher in active LN group compared to the other two groups (*P*-value <0.001); however, no statistically significant difference between the control and lupus non-nephritis group (*P*-value = 0.537). The lupus non-nephritis group showed significantly lower white blood cells and platelet counts in comparison to the other two groups (*P* < 0.001, 0.004 respectively and *P* < 0.001, 0.008 respectively). All cases of active LN showed consumed complement levels C3 and C4. Unlike the lupus non-nephritis group, only 9 cases had consumed complement levels. The ESR was significantly higher in the active LN group (*P*-value < 0.002). The 24-h urinary protein was significantly high in the active nephritis group (*P*-value < 0.001). There were significant differences between the 3 groups in urinary markers (uNGAL and uKIM-1) with the active LN group before treatment showing the highest levels (*P*-value < 0.001).

**Table 1:** Personal, clinical, and laboratory data of the study group

	Control (*n*= 20)	Lupus non-nephritis (*n*= 20)	Lupus nephritis at baseline (*n*= 20)	*P*^1^	*P*^2^	*P*^3^	*P*^4^
	No. (%)	No. (%)	No. (%)				
Sex (female)	15 (75 %)	16 (80 %)	16 (80 %)	*0.906*	*1.000*	*1.000*	*1.000*
Age (years)	30.30 ±7.79	32.35 ± 9.19	27.50 ± 6.31	*0.156*	*0.412*	*0.264*	*0.056*
SLEDAI score “10^5^”	0.00 ± 0.00	7.55 ± 4.71	18.50 ± 3.56	*0.000*	*0.000*	*0.000*	*0.000*
S. creatinine (mg/dl)	0.90 ± 0.22	0.94 ± 0.15	2.05 ± 1.37	*0.537*	*0.000*	*0.000*	*0.001*
ANA (folds)	0.00 ± 0.00	3.10 ± 1.48	4.10 ± 1.74	*0.000*	*0.000*	*0.000*	*0.074*
Anti dsDNA (fold)	0.00 ± 0.00	3.20 ± 1.24	4.95 ± 4.19	*0.000*	*0.000*	*0.000*	*0.115*
C3, C4, No. (%)							
— Normal	-	9 (45.0 %)	0 (0.0 %)	*0.001*	*-*	*-*	*0.001*
— Decreased	-	11 (55.0 %)	20 (100.0 %)				
HB (g/dl)	11.52 ± 1.25	8.43 ± 0.82	7.99 ± 1.64	*0.000*	*0.000*	*0.000*	*0.276*
WBCs × 10^3^/mm^3^	7.17 ± 1.53	3.62 ± 1.53	5.64 ± 2.02	*0.000*	*0.000*	*0.004*	*0.001*
PLT × 10^3^/mm^3^	274.30 ± 96.97	103.05 ± 63.25	197.05 ± 89.23	*0.000*	*0.000*	*0.008*	*0.000*
ESR (ml/s)	13.50 ± 5.71	87.00 ± 14.64	102.40 ± 25.45	*0.000*	*0.000*	*0.000*	*0.002*
24-h urinary protein (mg/dl)	68.00 ± 26.38	279.00 ±115.75	3132.00 ±2095.72	*0.000*	*0.000*	*0.000*	*0.000*
uNGAL (ng/ml)	13.35 ± 2.96	41.80 ± 9.28	71.25 ± 10.12	*0.000*	*0.000*	*0.000*	*0.000*
NGAL/creatinine	30.62 ± 7.21	61.95 ± 17.84	170.90 ± 38.37	*0.000*	*0.000*	*0.000*	*0.000*
uKIM-1 (ng/ml)	1.35 ± 0.19	1.61 ± 0.12	1.88 ± 0.19	*0.000*	*0.000*	*0.000*	*0.000*
KIM-1/creatinine	3.05 ± 0.38	2.40 ± 0.55	4.46 ± 0.87	*0.000*	*0.002*	*0.000*	*0.000*

*P*-value 1: the significance between control and lupus non-nephritis group, *P*-value 2: the significance between control and lupus nephritis group, *P*-value 3: the significance between lupus non-nephritis and *lupus* nephritis, *P*-value 4: the significance between the 3 groups

*ANA* antinuclear antibodies, *anti dsDNA* anti-double-stranded DNA, *HB* hemoglobin, *WBCs* white blood cells, *PLT* platelets, *ESR* erythrocyte sedimentation rate, *CRP* C-reactive protein, *SLEDAI* systemic lupus erythematous daily activity index

**Fig. 1:**
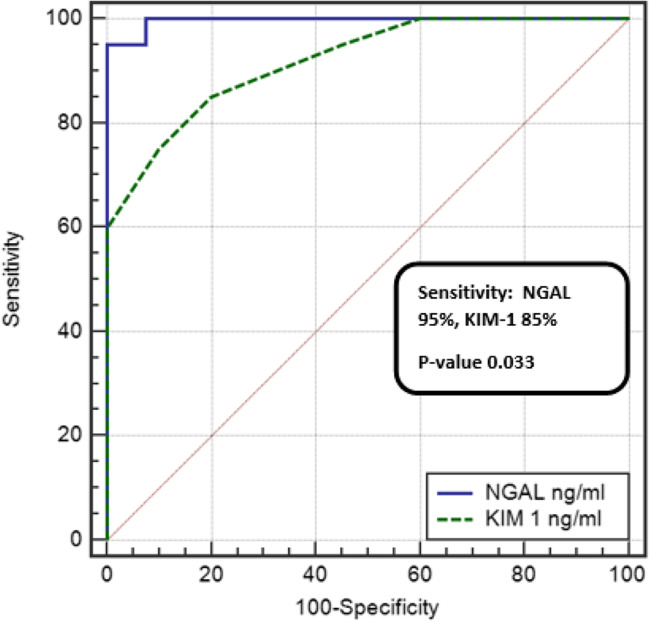
Pairwise comparison of ROC curves between uNGAL and uKIM-1 as predictors of lupus nephritis

**Fig. 2:**
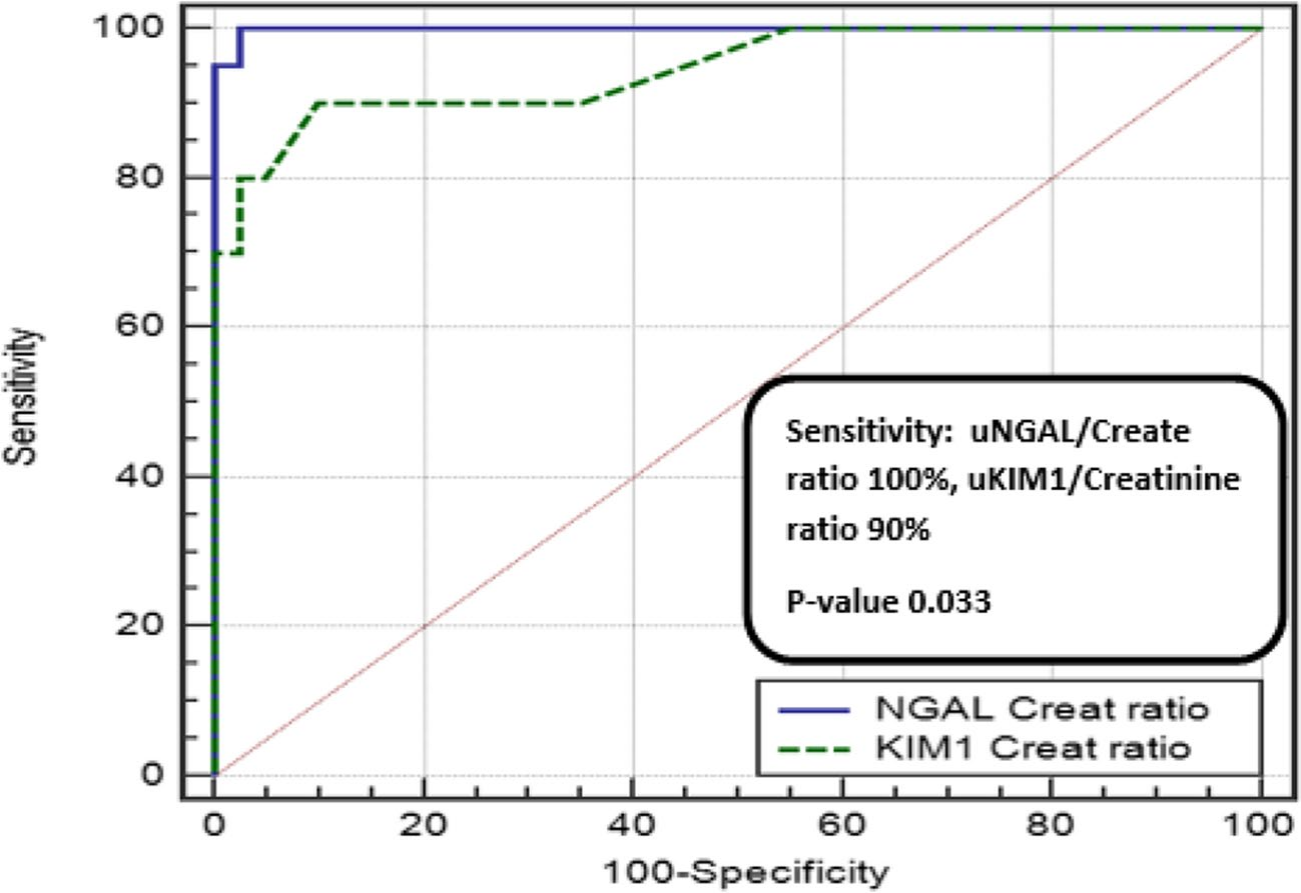
Pairwise comparison of ROC curves between NGAL/creatinine ratio and KIM-1/creatinine ratio as predictors of lupus nephritis

ROC curve analysis was used to establish the best cutoff values of each marker by measuring their sensitivity (true positive rate) and specificity (false positive rate). The analysis showed that a uNGAL level above 59 had a 95 % sensitivity and 100% specificity in the ability of diagnosis of active nephritis with an area under the curve (AUC) of 0.996. In addition, the uKIM-1 ROC analysis showed that at a level more than 1.6, it had an 85% sensitivity and 80% specificity with an area under curve of 0.919. The pairwise comparison of the two ROC analysis turned that uNGAL is the best predictor for LN as it had a higher sensitivity (95 vs 85%), a higher specificity (100 vs 80%), and a bigger area under curve (0.996 vs 0.919) (Fig. 1)(Fig. 2).

### After treatment

There was no significant difference in the group before and after treatment regarding serum creatinine and platelet count (*P*-value 0.462, 0.654), while there were significant differences in the SLEDAI score (*P*-value < 0.001), inflammatory markers, and serological markers (*P*-value < 0.001) (Table 2). In addition, uNGAL and uKIM-1 were significantly lower after treatment (*P*-value < 0.001).

**Table 2:** Comparison of laboratory investigation and clinical score before and after treatment in the lupus nephritis group

	(*n*= 20)	Lupus nephritis after 6 months (*n*= 20)	*P*
SLEDAI score “10^5^”	18.50 ± 3.56	5.80 ± 3.71	0.000
S. creatinine (mg/dl)	2.05 ± 1.37	2.19 ± 2.20	0.462
HB (g/dl)	7.99 ± 1.64	9.72 ± 1.46	0.001
WBCs ×10^3^/mml	5.64 ± 2.02	8.90 ± 3.72	0.001
PLT × 10^3^/mml	197.05 ± 89.23	175.00 ± 54.53	0.654
ESR (ml/s)	102.40 ± 25.45	66.25 ± 24.60	0.001
CRP (mg/dl)	12.90 ± 7.61	3.30 ± 6.30	0.001
ANA (fold)	4.10 ± 1.74	1.30 ± 0.47	0.000
Anti dsDNA (fold)	4.95 ± 4.19	0.55 ± 0.89	0.000
C3, C4, No. (%)			
— Normal	0 (0.0%)	15 (75.0%)	0.000
— Decreased	20 (100.0%)	5 (25.0%)	
24-h urinary proteins (mg/day)	3132.00 ± 2095.72	977.55 ± 433.19	0.000
uNGAL ng/ml	71.25 ± 10.12	36.45 ± 5.45	0.000
NGAL creatinine (ng/mg)	170.90 ± 38.37	61.03 ± 15.45	0.000
uKIM-1 ng/ml	1.88 ± 0.19	1.50 ± 0.19	0.000
KIM-1/creatinine (ng/mg)	4.46 ± 0.87	2.64 ± 0.88	0.000

*ANA* antinuclear antibodies, *anti dsDNA* anti-double-stranded DNA, *HB* hemoglobin, *WBCs* white blood cells, *PLT* platelets, *ESR* erythrocyte sedimentation rate, *CRP* C-reactive protein, *SLEDAI* systemic lupus erythematous daily activity index

### Correlations of the urinary markers with immunological markers

There was no significant correlation between the urinary markers before and after treatment with either clinical, inflammatory, activity and chronicity index, or serological marker of lupus nephritis (Table 3). That was with the except of the significant correlation between uNGAL, KIM-1/creatinine ratio, and WBCs.

**Table 3:** Correlation between urinary activity markers and laboratory investigations and clinical score in the studied group

	Lupus nephritis at baseline				Lupus nephritis after 6 months			
	uNGAL	uNGAL/Creat ratio	uKIM-1	uKIM-1/Creat ratio	uNGAL	uNGAL/Creat ratio	uKIM-1	uKIM-1/Creat ratio
S. creatinine (mg/dl)	*r* 0.324	*r* 0.076	*r* −0.153	*r* −0.086	*r* −0.216	*r* 0.193	*r* 0.025	*r* 0.117
	*P* 0.163	*P* 0.750	*P* 0.519	*P* 0.719	*P* 0.361	*P* 0.415	*P* 0.915	*P* 0.622
ANA (folds)	*r* 0.144	*r* 0.256	*r* −0.053	*r* 0.042	*r* −0.418	*r* −0.104	*r* 0.183	*r* 0.019
	*P* 0.544	*P* 0.277	*P* 0.824	*P* 0.859	*P* 0.067	*P* 0.662	*P* 0.440	*P* 0.937
Anti dsDNA (folds)	*r* 0.113	*r* 0.578	*r* −0.279	*r* 0.319	*r* −0.199	*r* 0.220	*r* 0.077	*r* 0.214
	*P* 0.636	*P* 0.008	*P* 0.234	*P* 0.171	*P* 0.400	*P* 0.352	*P* 0.746	*P* 0.365
HB (g/dl)	*r* −0.438	*r* −0.192	*r* 0.015	*r* 0.126	*r* 0.518	*r* 0.338	*r* −0.403	*r* 0.034
	*P* 0.053	*P* 0.418	*P* 0.950	*P* 0.597	*P* 0.019	*P* 0.145	*P* 0.078	*P* 0.885
WBCs × 10^3^/mm^3^	*r* −0.533	*r* 0.126	*r* 0.112	*r* 0.481	*r* −0.023	*r* 0.059	*r* 0.167	*r* 0.092
	*P* 0.015	*P* 0.596	*P* 0.637	*P* 0.032	*P* 0.923	*P* 0.806	*P* 0.483	*P* 0.701
PLT × 10^3^/mm^3^	*r* −0.074	*r* −0.305	*r* 0.367	*r* −0.016	*r* 0.167	*r* −0.176	*r* 0.159	*r* 0.115
	*P* 0.756	*P* 0.191	*P* 0.111	*P* 0.946	*P* 0.483	*P* 0.458	*P* 0.503	*P* 0.631
ESR (ml/s)	*r* 0.152	*r* 0.166	*r* 0.102	*r* 0.144	*r* −0.304	*r* 0.017	*r* −0.017	*r* 0.064
	*P* 0.524	*P* 0.485	*P* 0.669	*P* 0.546	*P* 0.193	*P* 0.944	*P* 0.942	*P* 0.787
CRP (mg/dl)	*r* 0.346	*r* 0.265	*r* 0.162	*r* 0.202	*r* −0.281	*r* 0.012	*r* 0.031	*r* 0.049
	*P* 0.136	*P* 0.259	*P* 0.496	*P* 0.392	*P* 0.231	*P* 0.960	*P* 0.896	*P* 0.838
24-h urinary protein (mg/day)	*r* 0.264	*r* −0.307	*r* 0.099	*r* −0.377	*r* −0.102	*r* 0.077	*r* −0.040	*r* −0.114
	*P* 0.261	*P* 0.188	*P* 0.678	*P* 0.101	*P* 0.667	*P* 0.747	*P* 0.868	*P* 0.632
SLEDAI score	*r* 0.085	*r* −0.130	*r* 0.295	*r* −0.109	*r* −0.240	*r* 0.161	*r* −0.170	*r* −0.026
	*P* 0.721	*P* 0.584	*P* 0.207	*P* 0.647	*P* 0.307	*P* 0.498	*P* −0.026	P 0.912
Activity index	*r* −0.388	*r* −0.132	*r* −0.071	*r* 0.049	-	-	-	-
	*P* 0.091	*P* 0.579	*P* 0.767	*P* 0.837	-	-	-	-
Chronicity index	*r* 0.150	*r* 0.069	*r* −0.274	*r* −0.063	-	-	-	-
	*P* 0.528	*P* 0.772	*P* 0.243	*P* 0.793	-	-	-	-

*ANA* antinuclear antibodies, *anti dsDNA* anti-double-stranded DNA, *HB* hemoglobin, *WBCs* white blood cells, *PLT* platelets, *ESR* erythrocyte sedimentation rate, *CRP* C-reactive protein, SLEDAI systemic lupus erythematous disease activity index

## Discussion

The main findings of our study are that (1) SLE patients had significantly higher uNGAL and uKIM-1 levels before treatment compared to control, with the active LN group showing the highest levels; (2) active LN patients had significantly higher SLEDAI score, activity serological markers (ESR, ANA, and anti-ds DNA), 24-h urinary protein, uKIM-1, and uNGAL; (3) uNGAL level above 59 had a 95% sensitivity and a 100% specificity in detecting LN, while uNGAL/creatinine ratio above 92 had a 100% sensitivity and a 97% specificity; (4) uKIM-1 level >1.6 had an 85% sensitivity and an 80% specificity in detecting LN, while uKIM-1/creatinine ratio had a 90 % sensitivity and 90% specificity at a level of more than 3.4; and (5) none of these urinary markers had significant correlations with either SLEDAI score, activity serological markers, or urinary protein before and after treatment except for a significant positive correlation between the uNGAL/creatinine ratio before treatment with the ANA. Moreover, there was no correlation between the urinary markers with either the pathological activity or the chronicity indices.

uNGAL, a 25 -kDa protein, is a member of the lipocalin superfamily that has been widely studied in patients of acute kidney injury. Pitashny et al. were the first to conclude that urinary lipocalin-2 is a potential marker for renal involvement in adult patients with SLE [16]. Consequent studies repeatedly showed that patients with renal flare have high levels of uNGAL in the SLE group compared to control [17, 26–29]. Our results clearly showed that uNGAL is a highly sensitive marker for renal involvement in lupus patients with active LN.

The role of KIM-1 as a tubulointerstitial marker has been previously proposed [30]. Subsequently, the tubular (t)-Kim-1 expression has been found to be specific to the ongoing tubular cell damage and dedifferentiation. Currently, the urinary (u)-KIM-1 is considered a marker for tubular damage [31]. However, its role as a marker for glomerular assessment in lupus nephritis was still questionable. In our study, the uKIM-1 was significantly higher in the active LN group. This comes in agreement with the current literature [24, 29, 32].

In terms of clinical score, we did not find a significant correlation between uNGAL and the total SLEDAI score. Previous studies had heterogenous results regarding this issue. Some studies showed a significant correlation between uNGAL with renal SLEDAI (rSLEDAI) and concluded its valuable use as a predictor of renal involvement [16, 33, 34]. However, other concluded that uNGAL is a strong predictor of rSLEDAI score but was not a strong predictor for total SLEDAI score [20, 35, 36]. Renal SLE disease activity index (rSLEDAI) is used to assess kidney disease activity. The score consists of the four kidney-related parameters: hematuria, pyuria, proteinuria, and urinary casts. Scores range from 0 (inactive renal disease) to a maximum of 16. This finding may refer to the fact that uNGAL is a marker for renal damage caused by non-immunological diseases.

Studies that investigated the association between uKIM-1 and clinical activity also showed variable results. Nazari et al. concluded that uKIM-1 correlated significantly with the total SLEDAI and the rSLEDAI score [32]. On the other hand, Nozaki et al.’s study showed no correlation between the uKIM-1 with either the total or the renal SLEDAI score [24].

The current study did not show any significant correlation between the uNGAL and the uNGAL/creatinine ratio to either serological or the pathological activity markers of LN. Other studies showed same results in both adults and children [37] with larger sample size [8] .

In terms of uKIM-1, Nozaki et al. found that uKim-1 level was significantly correlated with both proteinuria and tubular damage. In addition, uKim-1 level was highly associated with eGFR at baseline, and with serum creatinine after 6–8 months of treatment; however, there was no correlation between the uKim-1 level with either the urinary protein/creatinine ratio at baseline and after 6–8 months of treatment, anti-dsDNA; complement C4, nor the SLEDAI score [24]. Moreover, a study by Khadijah et al. showed a positive significant correlation between the uKIM-1 and anti-dsDNA, uProt/uCreat ratio, the total SLEDAI, and the rSLEDAI score. Meanwhile, there was an inverse significant correlation between uKIM-1 and serum C3 levels and insignificant inverse correlation with serum C4 levels [32].

The small sample size may underpower our study to detect any a significant correlation between the uKIM-1 and other activity markers either before or after treatment.

In the current study, the follow-up of the urinary biomarkers after treatment showed a significant decrease in these biomarkers after treatment apart from serum creatinine which had an insignificant difference before and after treatment. This could be attributed to the outcome of our study active LN group in which only one case had complete response while the rest of lupus LN either had partial or no response. Many studies referred to the uNGAL and the uKIM-1 as good predictors for lupus LN activity changes. In juvenile SLE patients, both urine monocyte chemoattractant protein-1 (uMCP1) and serum C3 can indicate renal involvement, while both uMCP1 and uNGAL can predict subsequent renal disease activity changes [38]. Other studies showed that uNGAL can differentiate between active and inactive LN [34, 39]. The same finding was proven as regard uKIM-1 as predictors for changes in lupus nephritis activity [24].

## Limitations and recommendation

Because of financial issues, we had a small size sample for each group which may underpower the correlations analysis. In addition, the study was mono-center and mono-ethnic one. We recommend conducting multiethnic multi-center study with a larger sample size and a longer follow-up duration.
